# Characterization of cereblon-dependent targeted protein degrader by visualizing the spatiotemporal ternary complex formation in cells

**DOI:** 10.1038/s41598-020-59966-5

**Published:** 2020-02-20

**Authors:** Tomohiro Kaji, Hiroshi Koga, Mutsumi Kuroha, Toshihiko Akimoto, Kenji Hayata

**Affiliations:** Biochemistry Research Group, Biological Research Department, Daiichi Sankyo RD Novare Co., Ltd., 1-16-13 Kitakasai, Edogawa-ku, Tokyo 134-8630 Japan

**Keywords:** Imaging, Cellular imaging, Proteolysis, Biological techniques, Cell biology, Drug discovery, Drug screening

## Abstract

Targeted protein degradation (TPD) through a proteasome-dependent pathway induced by heterofunctional small molecules is initiated by the formation of a ternary complex with recruited E3 ligases. This complex formation affects the degradation ability of TPD molecules, and thus we tested for visualization of the intracellular dynamics of ternary complex formation. In this study, we applied the fluorescent-based technology detecting protein-protein interaction (Fluoppi) system, in which detectable fluorescent foci are formed when ternary complex formation induced by TPD molecules occurs in cells. We show here that cells coexpressing BRD4 and cereblon (CRBN) tagged with the Fluoppi system formed detectable foci in both live and fixed cells only when treated with BRD4-targeting degraders utilizing CRBN as an E3 ligase in dose- and time-dependent manners. Notably, the maintenance and efficacy of TPD molecule-induced foci formation correlated with the ability to degrade target proteins. Furthermore, we demonstrated that BRD4-targeting and FKBP12^F36V^-targeting degraders formed ternary complexes mainly in the nucleus and cytoplasm, respectively, suggesting that TPD molecules utilize the proteasome to degrade target proteins in their corresponding localized region. Our results also suggest that the Fluoppi system is a powerful tool for characterizing TPD molecules by visualizing the spatiotemporal formation of ternary complex.

## Introduction

Removing a protein of interest such as a disease-causative undruggable protein through targeted protein degradation (TPD) is anticipated to become a new therapeutic modality^1–4^. TPD molecules are bifunctional molecules composed of target-binding and E3 ligase-binding components connected by a linker, and these three moieties have been refined in order to augment the abilities of TPD molecules and thereby discover efficacious new drugs^5–7^.

To confirm the potency of TPD molecules *in vitro*, the capacities not only to degrade the target proteins in cells, but also to induce a ternary complex with a target protein, a TPD molecule and an E3 ligase, are often examined. Because the ternary complex formation initiates the ubiquitination of target proteins followed by their degradation in cells, it is important to analyze the ternary complex formation in order to improve the design of TPD molecules, potentially leading to efficacious new drugs^8–10^. For this purpose, binding assays such as amplified luminescent proximity homogeneous assay (AlphaLISA)^11^ and surface plasmon resonance (SPR)^12^ techniques have been performed in cell-free systems. Furthermore, a recent report described the measurement of bromodomain and extra-terminal (BET) protein degraders’ capacity for ternary complex formation or ubiquitination in cells using NanoBRET systems^13^ and that time-lapse protein-protein interaction imaging via the separation of phases-based protein interaction reporter (SPPIER) visualized early ternary complex formation induced by BET protein degraders^14^. Even if these techniques can measure the ternary complex formation ability, it is difficult to clarify the maintenance and efficacy of intracellular spatiotemporal dynamics of ternary complex formation induced by TPD molecules. Therefore, we utilize the fluorescent-based technology detecting protein-protein interaction (Fluoppi) system to clarify such dynamics in cells.

The Fluoppi system is a tool for detecting protein-protein interactions in live and fixed cells^15–17^. Briefly, it involves the coexpression of an Azami-Green (AG) fused protein and another Assembly helper (Ash)-tagged protein in cells. AG-derived fluorescence is uniformly distributed in the absence of interaction. However, once the interaction occurs, AG-derived fluorescence forms detectable foci because of the excess assembly of two proteins through Ash tags.

In this paper, we present data showing not only that TPD molecules form specific foci with the Fluoppi system, which enables determination of where and when the TPD molecules induce ternary complexes in cells, but also that the duration of foci formation is correlated with the degradation abilities of TPD molecules.

## Results

### Cereblon (CRBN)-dependent BET protein degraders induce specific foci formation with the Fluoppi system

**Figure 1:**
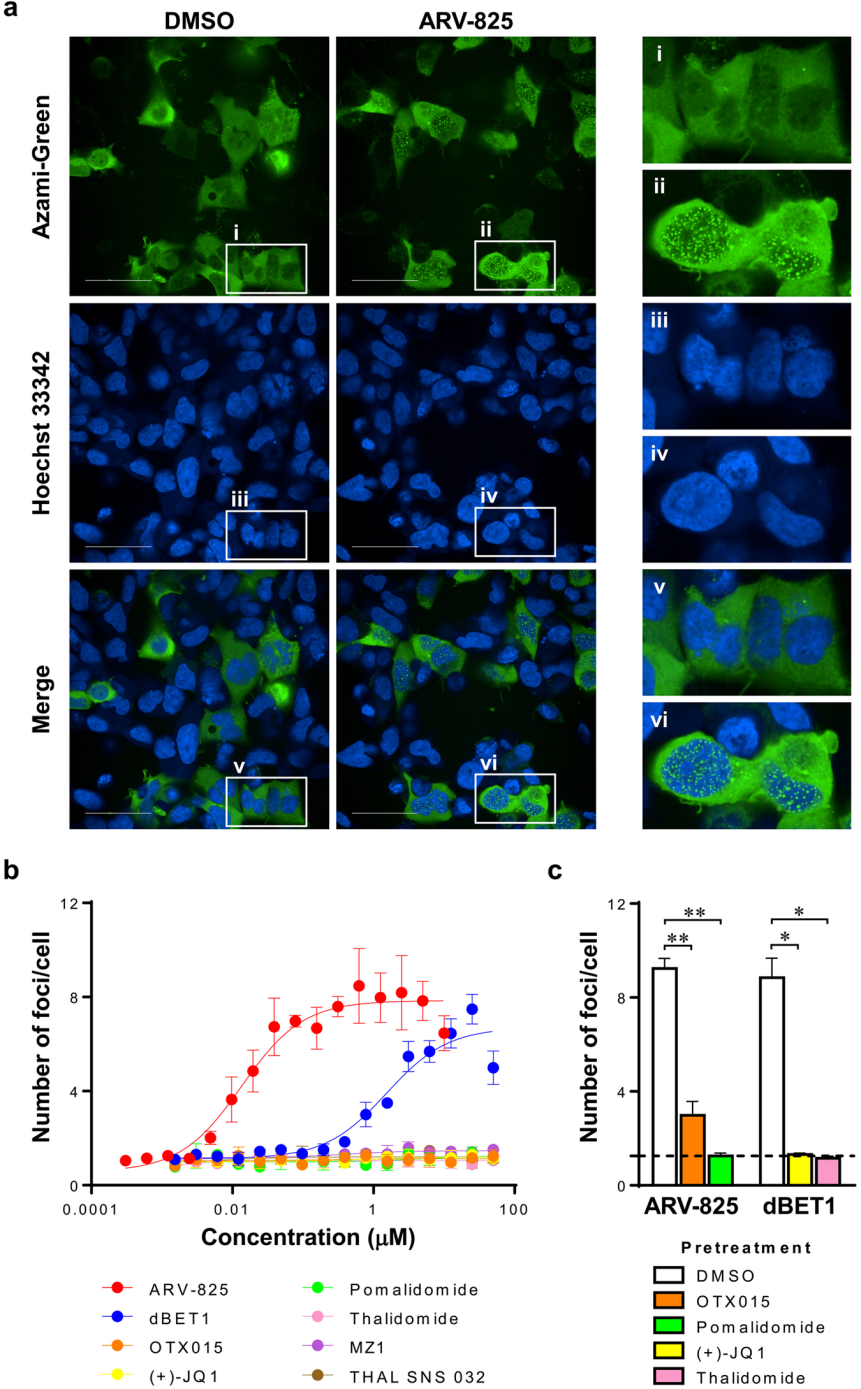
TPD molecules induced specific foci in a dose-dependent manner with the Fluoppi system. 293A cells coexpressing BRD4-Ash and CRBN-AG fusion proteins were prepared. (**a**) Cells were treated with 0.5% DMSO (left panels) or 0.1 µM ARV-825 (middle panels) for 30 min. Cells were fixed with formalin containing Hoechst 33342, followed by confocal microscopic analysis. AG (green, left panels), Hoechst 33342 (blue, middle panels) and merged images (right panels) are shown. Numbered magnified areas highlighted with white boxes (i–vi) are shown in the right panels. Data shown are representative of five independent experiments. Scale bar indicates 50 µm. (**b**) Cells were treated with different concentrations of ARV-825 (red), dBET1 (blue), OTX015 (orange), (+)-JQ1 (yellow), pomalidomide (green), thalidomide (pink), MZ1 (purple) and THAL SNS 032 (brown) in 0.5% DMSO for 30 min. Cells were fixed with formalin containing Hoechst 33342, followed by confocal microscopic analysis. The numbers of foci formed by AG and Hoechst 33342-positive cells were quantified and the number of foci per cell was calculated. EC_50_ values of ARV-825 and dBET1 were 0.013 and 1.5 µM, respectively. Data shown are the mean (n = 4) ± S.D. and are representative of two independent experiments. (**c**) Cells were treated with vehicle (open), BRD4 binder [10 µM OTX015 (orange) or 30 µM (+)-JQ1 (yellow)] and CRBN binder [50 µM pomalidomide (green) or 250 µM thalidomide (pink)] in 0.5% DMSO for 1 h prior to treatment with TPD molecules (0.1 µM ARV-825 or 10 µM dBET1) in 0.5% DMSO for 30 min. The number of foci per cell was determined as in (**b**). The dotted line indicates the number of foci per cell from cells pretreated and treated with vehicle (DMSO) only. Data shown are the mean (n = 4) + S.D. and are representative of two independent experiments. *p < 0.001, **p < 0.0001.

To assess the possibility that the Fluoppi system could be applied to evaluate TPD molecules, we first chose ARV-825^18^, which was composed of BRD4-binding OTX015^19^ and CRBN-binding pomalidomide^20^ and degraded BRD4 by recruiting CRBN as an E3 ligase. We constructed Fluoppi expression vectors coding BRD4 fused to the Ash tag at the C-terminus (BRD4-Ash) and CRBN fused to AG at the C-terminus (CRBN-AG), and cotransfected these vectors into 293A cells. We then treated these cells with 0.5% DMSO or 0.1 µM ARV-825 for 30 min and fixed them, followed by confocal microscopic analysis. As shown in Fig. 1a, ARV-825 induced specific foci mainly in the nucleus, but DMSO did not. Next, we treated these cells with various compounds and determined the number of foci per cell. Figure 1b indicates that only TPD molecules, ARV-825 and dBET1^11^, which bound both BRD4 and CRBN, induced foci formation in a dose-dependent manner. This suggests that neither parts of TPD molecules such as BRD4-binding (+)-JQ1^21^, OTX015, CRBN-binding thalidomide^22^ and pomalidomide nor TPD molecules such as MZ1^23^ bound to BRD4 and von-Hippel-Lindau (VHL) and THAL SNS 032^24^ bound to CDK9 and CRBN form specific foci. Furthermore, to confirm the specificity of TPD molecule-induced foci formation, cells were treated with an excess amount of the moieties of ARV-825 or dBET1 prior to the treatment with these TPD molecules (Fig. 1c). As expected, pretreatment with the parts of TPD molecules remarkably reduced the foci formation, suggesting that the foci induced by TPD molecules represent the ternary complex formation. We also checked the opposite combination of Fluoppi tags, with the results showing that AG-fused BRD4 formed foci with its binders even in the absence of Ash-fused CRBN (Supplementary Fig. S1), suggesting that AG-fused BRD4 could nonspecifically form the foci when treated with BRD4 binders (see Discussion).

### Foci formed in the Fluoppi system are time-dependent and represent ternary complexes to be degraded by proteasome

**Figure 2:**
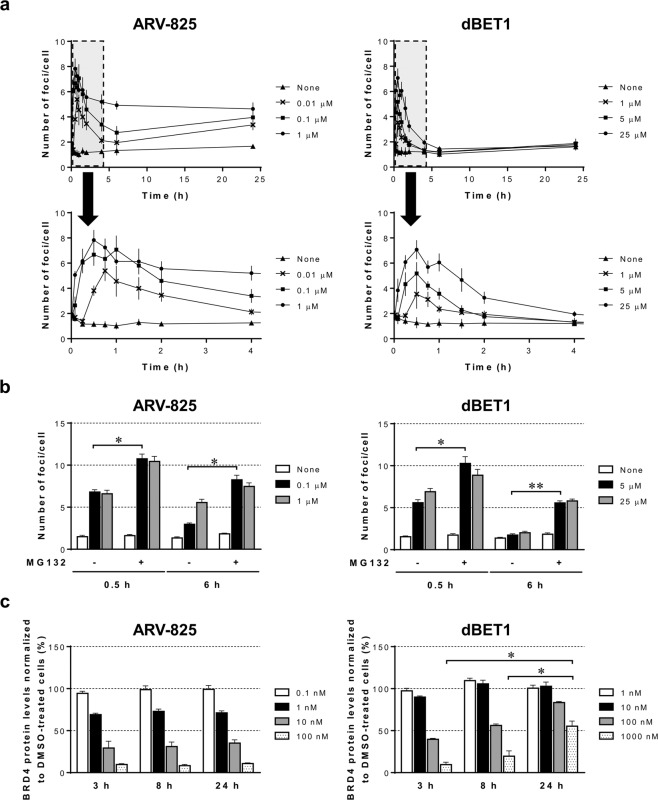
ARV-825 sustained foci formation and BRD4 protein degradation longer than dBET1 in a proteasome-dependent manner. (**a**) 293A cells prepared as in Fig. 1 were treated with ARV-825 (0.01, 0.1 or 1 µM, left panels) or dBET1 (1, 5 or 25 µM, right panels) in 0.5% DMSO for the indicated time. Upper panels show the time course of foci formation until 24 h. Magnified areas of the early period highlighted with gray dashed boxes in upper panels are also shown in lower panels. The number of foci per cell was determined as in Fig. 1b. Data shown are the mean (n = 4) ± S.D. and are representative of three independent experiments. (**b**) 293A cells prepared as in Fig. 1 were treated with or without 2 µM MG132 in 0.1% DMSO for 1 h prior to the stimulation with 0.5% DMSO, 0.1 or 1 µM ARV-825, or 5 or 25 µM dBET1 for 0.5 or 6 h. The number of foci per cell was determined as in Fig. 1b. Data shown are the mean (n = 4) + S.D. and are representative of two independent experiments. *p < 0.001, **p < 0.0001. (**c**) 293A cells with knock-in of the HiBiT tag in the BRD4 gene at the N-terminus were cultured on a 384-well plate and stimulated with 0.1% DMSO, ARV-825 (0.1, 1, 10 or 100 nM, left panel) or dBET1 (1, 10, 100 or 1000 nM, right panel) for 3, 8 or 24 h. BRD4 expression levels were monitored with HiBiT-derived luminescent signals and normalized to the DNA contents in each well. The percentages of DMSO-treated cells are shown as the mean (n = 4) + S.D. Data shown are representative of two independent experiments. *p < 0.001.

Next, we investigated the time course of foci formation induced by ARV-825 or dBET1. As shown in Fig. 2a, both molecules induced foci as early as 5 min after stimulation and the levels of foci formation peaked within 1 h. Intriguingly, foci induced by dBET1 were diminished in 6 h, whereas ARV-825 maintained foci formation 24 h later, suggesting that dBET1 could not keep the ternary complex in cells for longer than 6 h. Previous reports suggest that the proteasome inhibitor MG132 blocks targeted protein degradation by TPD molecules^13,18^. Therefore, we pretreated cells cotransfected with BDR4-Ash and CRBN-AG with MG132 before the stimulation with ARV-825 or dBET1 and investigated the formation of foci (Fig. 2b). Surprisingly, inhibition of protein degradation through the proteasome sustained or upregulated foci formation, even after the treatment with dBET1 for 6 h. These results indicated that TPD molecules maintained the ternary complex in the absence of targeted protein degradation. To confirm that the foci formation correlates with the degradation of target proteins, we next checked the time-dependent protein degradation levels with these molecules. To this end, we utilized the HiBiT tag system, which easily enabled measurement of the endogenous protein expression levels in combination with the CRISPR/Cas9 knock-in system^25^. Cells harboring the HiBiT sequence in the N-terminus of the BRD4 gene were treated with ARV-825 or dBET1 for 3, 8 and 24 h (Fig. 2c). The obtained results show that ARV-825 continuously reduced the protein levels of BRD4 for 24 h, while dBET1 lost its degradation ability after 3 h, which was correlated with the rapid reduction of foci formation as shown in Fig. 2a and consistent with the observation that dBET1 was unstable when the proteasome pathway was active (Fig. 2b). Taken together, these results imply that the Fluoppi system detects the TPD molecule-induced ternary complex amenable to proteasomal degradation.

### Fluoppi analysis reveals where ternary complex formation occurs in cells

**Figure 3:**
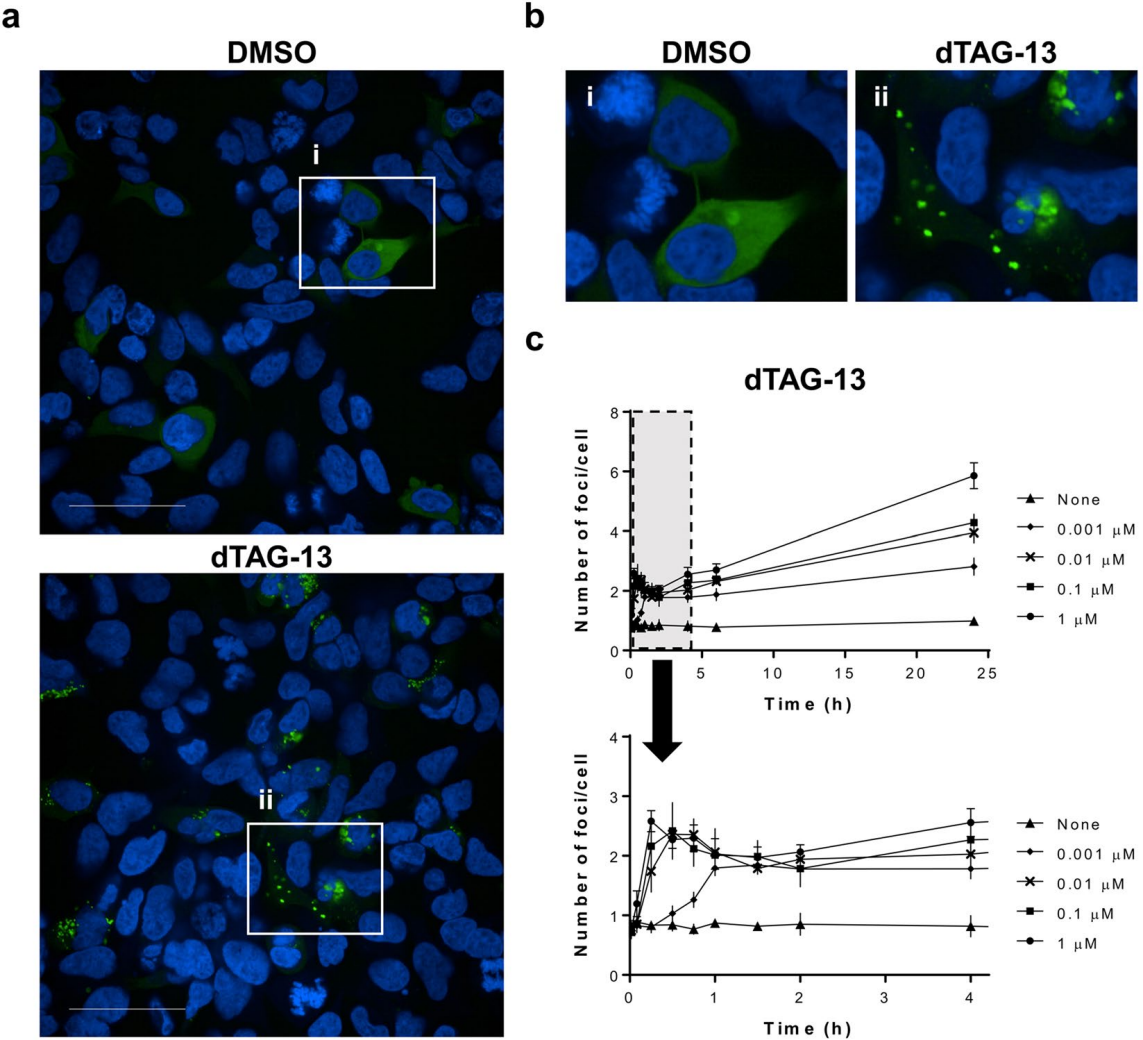
dTAG-13 continuously induced the ternary complex mainly in the cytoplasm. 293A cells coexpressing Ash-FKBP12^F36V^ and CRBN-AG fusion proteins were prepared. (**a**) Cells were treated with 0.1% DMSO (upper) or 0.1 µM dTAG-13 (lower) for 1 h. Cells were fixed with formalin containing Hoechst 33342, followed by confocal microscopic analysis. AG (green) and Hoechst 33342 (blue) images were merged. Data shown are representative of three independent experiments. Scale bar indicates 50 µm. (**b**) Numbered magnified areas (i, ii) highlighted with white boxes in (**a**) are shown. (**c**) Cells were treated with dTAG-13 (0.001, 0.01, 0.1 or 1 µM) in 0.1% DMSO for the indicated time. Upper panel shows the time course of foci formation until 24 h. Magnified area of the early period highlighted with a gray dashed box in the upper panel is also shown in the lower panel. The number of foci per cell was quantified as in Fig. 1b. Data shown are the mean (n = 4) ± S.D. and are representative of two independent experiments.

BRD4 is a member of the BET family, which localizes in the nucleus^26^. In this paper, we show that the foci formation induced by BRD4 targeting degraders mainly occurred in the nucleus (Fig. 1a). We also tested another TPD molecule, dTAG-13^27,28^, which targets FKBP12 containing F36V mutation (FKBP12^F36V^) and located in the cytoplasm^29^ and binds CRBN as an E3 ligase. Cells coexpressing FKBP12^F36V^ fused to Ash tag at the N-terminus (Ash-FKBP12^F36V^) and CRBN-AG were treated with 1 µM dTAG-13 for 1 h and fixed, followed by confocal microscopic analysis (Fig. 3a,b). As expected, dTAG-13 induced specific foci mainly in the cytoplasm. We also investigated the time course of foci formation by dTAG-13 (Fig. 3c). The obtained results indicated that dTAG-13 continuously increased the foci formation until 24 h after treatment. In addition, ARV-825 continuously formed foci for 24 h, while dBET1 lost such formation after 6 h, as shown in Fig. 2a. Our results indicate that the occurrence and duration of foci formation are dependent on the properties of TPD molecules.

### Ternary complex formation occurs immediately after the treatment of TPD molecules

**Figure 4:**
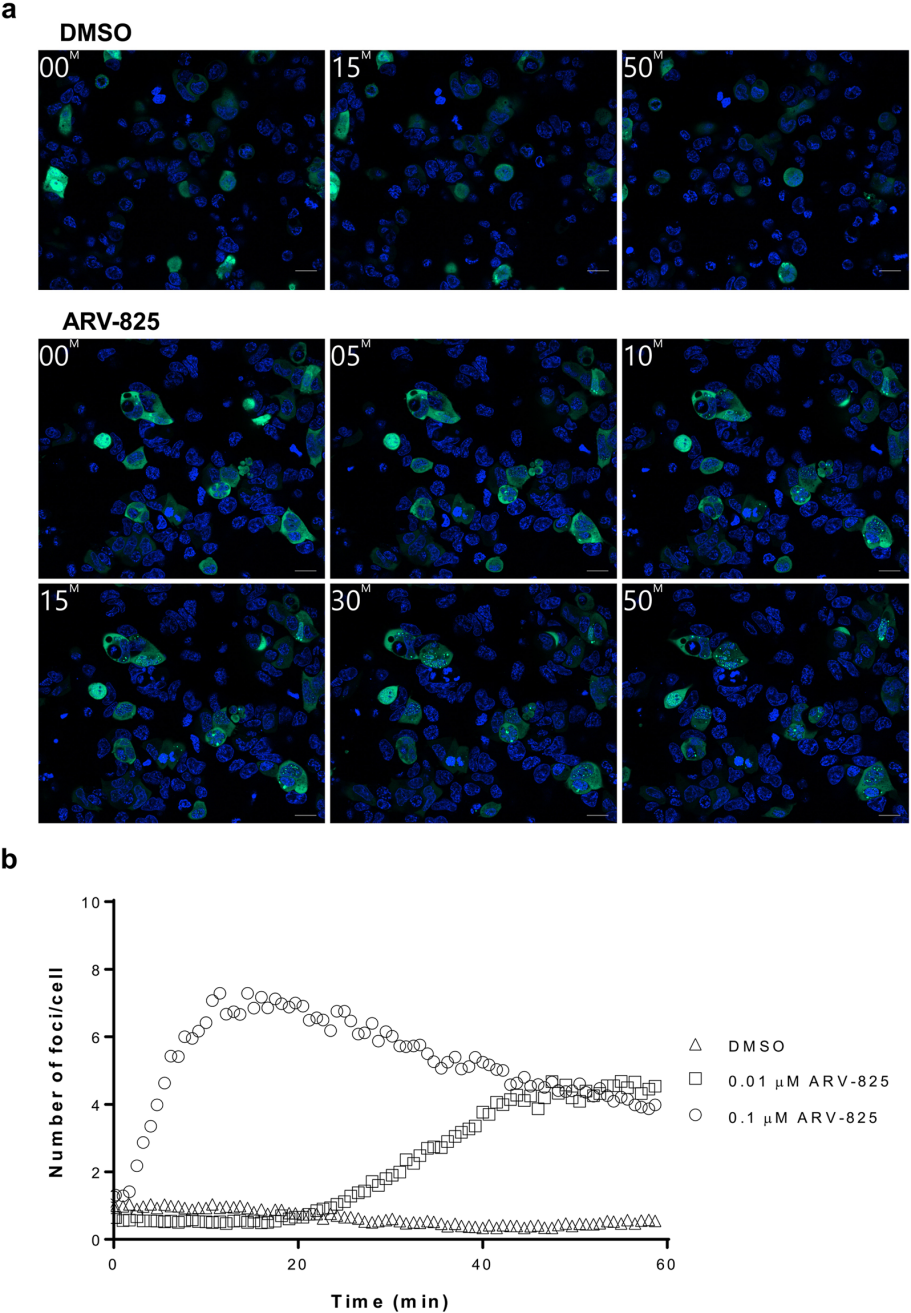
Early foci formation induced by ARV-825 by live-cell imaging. (**a**) 293A cells prepared as in Fig. 1 were stained with Hoechst 33342. After the addition of 0.05% DMSO (upper panels, from Supplementary Movie S1) or 0.1 µM ARV-825 (lower panels, from Supplementary Movie S2), live-cell imaging of AG (green) and Hoechst 33342 (blue) images was performed for 1 h with 45-s intervals. Time-lapse images at the indicated time (min) are shown. The delay between the injection of DMSO or ARV-825 and the first image acquisition (0 min) was approximately 80 s. Data shown are representative of three independent experiments. Scale bar indicates 20 µm. (**b**) Cells were treated with 0.05% DMSO (triangle, Supplementary Movie S1) and 0.1 (circle, Supplementary Movie S2) or 0.01 µM ARV-825 (square, Supplementary Movie S3). Each dot indicates the average number of foci in the nucleus per cell from three acquired images in each well. Data shown are representative of three independent experiments.

To visualize the formation of ternary complex at early time points after the addition of TPD molecules, we utilized real-time monitoring. Cells coexpressing BRD4-Ash and CRBN-AG were prepared for live-cell imaging, and AG and Hoechst 33342 images were monitored after the addition of DMSO or ARV-825 for 1 h (Fig. 4a,b and Supplementary Movies S1–S3). The obtained results showed that treatment with ARV-825 (0.1 µM) initiated the formation of foci in the nucleus within a few minutes and this peaked within 30 min, which is similar to the results depicted in Fig. 2a. However, treatment with a lower concentration of ARV-825 (0.01 µM) gradually increased foci formation, indicating that the early formation of ternary complexes induced by TPD molecules occurred in time- and dose-dependent manners.

## Discussion

TPD molecules link target proteins to E3 ligases to initiate ubiquitination of the target proteins, followed by degradation of the target proteins via a ubiquitin-proteasome dependent pathway. To date, TPD molecules’ abilities have been mainly evaluated in terms of capacities such as binding to target proteins and E3 ligases in cell-free systems^7,11^ or by cell-based BRET^13^ and SPPIER^14^ assays, or in terms of the potency for degrading target proteins in cells^5^. Because of the complexity of the cellular environment, it is also highly beneficial for the design of TPD molecules to monitor the dynamics of the ternary complex formation in cells^8,9^.

From the perspective of evaluating the intracellular ternary complex formation, the NanoBRET system has advantages for enabling the real-time monitoring of ternary complex formation biochemically with a high bioluminescent signal and for acquiring robust data because of the use of the acceptor/donor channel emission ratio as an evaluation criterion^13^. However, compared with fluorescent protein-based assays, such as the Fluoppi and SPPIER systems, the NanoBRET system has lower signal-to-noise ratios due to it being based on Förster resonance energy transfer (FRET) and it remains difficult to monitor the intracellular dynamics of the complex visually. One potential reason for this is the degradation of the target protein itself, resulting in loss of the energy donor or acceptor in the NanoBRET system, while fluorescent protein-based assays form fluorescent spots with assembled target proteins and the disruption of a target protein appears to have little effect on the foci formation. In addition to these advantages of robustness to the degradation of target proteins and high brightness with condensed fluorescent proteins, the SPPIER system can monitor two interacting proteins with different fluorescent proteins, which facilitates the understanding of individual molecular dynamics^14^. However, this approach has the disadvantages of being applicable only to live-cell imaging and that its spatial resolution still needs to be improved. In fact, SPPIER-tagged BRD4 appeared to be localized in the cytosol despite BRD4 being a nuclear protein^26^, which was probably due to huge protein aggregation or the specific characteristics of the fluorescent protein tags used in the SPPIER system. The Fluoppi system also utilizes the assembly of fluorescent proteins and, for this reason, has advantages similar to those of the SPPIER system in detecting the ternary complex. However, the Fluoppi system has better spatial resolution, such as enabling the intracellular localization of target proteins (see below), and also enables spatiotemporal visualization of ternary complex formation with both live and fixed cells, contributing to the further development of flexible assays; for example, fixed foci-formed cells can be subjected to staining with the antibodies against other proteins. However, the Fluoppi system has the disadvantage that it requires preliminary examinations to eliminate the possibility of nonspecific foci formation (see below).

Previous studies suggested that ARV-825 can more efficiently decrease the expression of BRD4 and the proliferation of leukemia cells than dBET1^30,31^. Our results also indicated that ARV-825 induced specific foci formation more effectively than dBET1 (Fig. 1b). It is conceivable that the difference in EC_50_ values of foci formation among TPD molecules reflects the ability to downregulate the target protein. It is supposed that the time course of foci formation by these two BET protein degraders was strongly correlated with maintenance of the levels of degradation of the target proteins (Fig. 2a,c). These results suggest a strong correlation between the level of time-dependent formation of ternary complex and the level of targeted protein degradation, supporting the notion that a stable ternary complex leads to efficient targeted protein degradation^8^. In addition, dBET1 has been reported to partially cancel the degradation of BRD4 upon the treatment of cells for 24 h due to the instability of the phthalimide component^11^, which is consistent with our observations (Fig. 2c). Because the inhibition of proteasome-dependent targeted protein degradation by a proteasome inhibitor sustained or increased the foci formation induced by dBET1 (Fig. 2b), the data obtained with Fluoppi analysis first revealed that the dissociation of dBET1 from the ternary complex by the proteasome-dependent degradation of BRD4 caused the instability of dBET1 in cells, resulting in the rapid loss of its degradation ability (Fig. 2a,c). Moreover, the Fluoppi system also allows us to monitor early foci formation in live cells (Fig. 4 and Supplementary Movies S1–S3), and we show that the peak time of foci formation after the addition of ARV-825 depends on its concentration (Fig. 4b), which is similar to the results obtained with other systems^13,14^. Collectively, these findings show that the Fluoppi system is a useful tool for evaluating the efficacy and longevity of TPD molecules in cells.

It has remained unclear where the TPD molecules form ternary complexes and degrade the target proteins. We utilized BRD4 as an example of a nuclear protein^26^ and FKBP12 as a cytoplasmic protein^29^, and clearly showed that BRD4-degrader and FKBP12^F36V^-degrader formed ternary complexes mainly in the nucleus (Fig. 1a and Fig. 4a) and cytoplasm (Fig. 3a,b), respectively. As previously reported, the ubiquitin-proteasome systems function in the cytoplasm and nucleus^32,33^; our results therefore clearly suggest that TPD molecules utilize the proteasome to degrade target proteins in their corresponding localized region. To summarize, our data presented in this report reveal that the Fluoppi system can characterize TPD molecules by investigating when and where ternary complex formation takes place.

The Fluoppi system enables the visualization of protein-protein interaction in the form of foci created by the accumulation of AG with Ash tags. Specific foci were induced by TPD molecules when appropriate target proteins and E3 ligases fused with Fluoppi tags were coexpressed (Fig. 1b); however, nonspecific foci formed when AG-fused protein accumulated alone in the presence of its binder (Fig. S1). We speculate that this formation of nonspecific foci with AG-fused BRD4 occurred for the following reasons. BRD4 binds the acetylated histone H3 and H4 in steady-state conditions^34^; however, its binding is disrupted by BET inhibitors such as (+)-JQ1 and OTX015, followed by the rapid accumulation of dissociated free BRD4 in the nucleus^18,21,30^. Moreover, BRD4 molecules interact with each other^35^, so it is conceivable that BET inhibitor-induced free AG-fused BRD4 molecules accumulate in the nucleus and associate with each other to form fluorescent foci even in the absence of Ash-tagged CRBN. It is also suggested by another report that tetrameric AG-fused oligomeric proteins could potentially form huge foci in cells^36^. Therefore, to avoid nonspecific foci formation in the study of TPD molecules with the Fluoppi system, it is important to confirm in advance that AG-fused protein cannot form foci by self-oligomerization and that TPD molecules or binders of target proteins or E3 ligases used as moieties of TPD molecules cannot induce foci in cells expressing AG-fused protein alone. In this study, we have confirmed that AG-fused CRBN could not form foci in the steady state or upon stimulation with CRBN binders such as pomalidomide or thalidomide (Fig. 1); therefore, the combination of AG-fused CRBN and Ash-tagged target protein can be applied for assessing various CRBN-dependent TPD molecules. We summarize the workflow for the evaluation of TPD molecules with the Fluoppi system in Supplementary Table S1.

Lastly, this Fluoppi system can be adapted for a readout method in high-throughput screening. Using a 384-well plate format, even though we performed both kinetic live-cell imaging (Fig. 4) and microscopic analysis of fixed cells (Fig. 1) with the transiently transfected cells, it is preferable to use cell lines stably expressing the Fluoppi-tagged proteins for high-throughput screening because the transfection efficiency in each experiment strongly affects the quantification of foci per cell. Given that the spatiotemporal visualization by the Fluoppi system enables detailed analysis of a TPD molecule’s potency, our approach amenable to high-throughput screening permits large-scale *in vitro* assessment, which would be highly valuable to the drug discovery process such as lead optimization. We have now investigated the ternary complex formation induced by TPD molecules recruiting another E3 ligase with the Fluoppi system.

TPD is an emerging technology with therapeutic applications and is rapidly developing in terms of its use for drug production. Therefore, multiple methods for characterizing TPD molecules will soon be needed, and our results provide a new option for evaluating TPD molecules’ properties by visualizing the spatiotemporal formation of ternary complex in cells.

## Methods

### Reagents

ARV-825, dBET1, (+)-JQ1, OTX015 and pomalidomide were purchased from Cayman. MZ1, THAL SNS 032, dTAG-13 and MG132 were purchased from Tocris Bioscience. Thalidomide was purchased from Sigma. All compounds were dissolved with DMSO (Merck Millipore).

### Fluoppi vector construction

Fluoppi vectors (phAG-MNL, pAsh-MNL or pAsh-MCL) were provided by Medical & Biological Laboratories. ORF cDNAs of human BRD4, FKBP12 or cereblon (CRBN) were obtained from Flexi ORF Clone (Promega). FKBP12^F36V^ single point mutation was inserted using PrimeSTAR Mutagenesis Basal Kit (Takara Bio), in accordance with the manufacturer’s instructions. ORFs amplified with PrimeSTAR Max DNA Polymerase (Takara Bio) were inserted into Fluoppi vectors using In-Fusion HD cloning kit (Takara Bio), in accordance with the manufacturer’s instructions.

### Cell preparation for Fluoppi analysis

293A cells (Thermo Fisher Scientific) were cultured with Dulbecco’s Modified Eagle’s Medium (Thermo Fisher Scientific) containing 10% FBS (HyClone), MEM Non-Essential Amino Acids Solution (Thermo Fisher Scientific) and GlutaMax (Thermo Fisher Scientific) within a humidified incubator with 5% CO_2_ at 37 °C. Cells were harvested and 5 × 10^5^ of them were plated on a six-well plate (Corning). The next day, phAG and pAsh vectors were cotransfected with Lipofectamine 3000 reagents (Thermo Fisher Scientific), in accordance with the manufacturer’s instructions. The ratios of phAG and pAsh vectors were as follows: phAG-MNL-CRBN and pAsh-MNL-BRD4 were used at a ratio of 3:1 for the BRD4/CRBN assay and phAG-MNL-CRBN and pAsh-MCL-FKBP12^F36V^ were used at a ratio of 9:1 for the FKBP12^F36V^/CRBN assay. The following day, cells were harvested and counted. A total of 2 × 10^4^ cells were plated on a 384-well plate (PerkinElmer) or frozen using Cell Banker (Wako) for live-cell imaging analysis.

### Microscopic analysis of fixed cells treated with compounds

Compounds and DMSO were directly added to cultured cells with a D300e digital dispenser (Tecan). In some experiments, cells were pretreated with MG132 or moieties of TPD molecules for 1 h before the treatment with TPD molecules. After incubation for the indicated times, cells were fixed with 20 µL of Mildform 20N (Wako) containing 1 µg/mL Hoechst 33342 (Sigma) for 15 min, followed by washing with 50 µL of PBS (Thermo Fisher Scientific) twice. After washing, the cells were immersed in 50 µL of PBS and the plate was sealed. AG and Hoechst 33342 images were acquired with Opera Phenix (PerkinElmer). Twenty-five fields of view per well were acquired using a ×63 water-immersed objective lens. The numbers of foci formed by AG and Hoechst 33342-positive cells were quantified and the number of foci per cell was calculated with Harmony 4.9 software (PerkinElmer). EC_50_ values of foci formation of TPD molecules were calculated with Prism 6 software (GraphPad Software).

### Kinetic live-cell imaging of ARV-825

Frozen cells were thawed and 2.5 × 10^4^ of them were plated on a 384-well plate. The next day, cells were washed with culture medium and incubated with 20 µL of culture medium containing 2 µg/mL Hoechst 33342 for more than 30 min. The culture plate was set into Cell Voyager 7000 S (CV7000S, Yokogawa) with a humidified 5% CO_2_ incubator at 37 °C. After the addition of 20 µL of culture medium containing 0.1% DMSO or 0.02 or 0.2 µM ARV-825 in 0.1% DMSO, three fields of view in a well were acquired with a ×60 water-immersed objective lens for 1 h at 45-s intervals. CellPathfinder software (Yokogawa) was used to create imaging movies and count the foci that formed.

### Measurement of intracellular BRD4 protein levels

293A cells harboring a HiBiT tag sequence^25^ in the N-terminus of the BRD4 gene (293A_HiBiT-BRD4) were established using the CRISPR-Cas9 system in our laboratory. Four thousand cells per well were plated on a 384-well white plate (Greiner). The next day, cells were treated with DMSO or BET protein degraders at the indicated concentrations and incubated for 3, 8 and 24 h. After incubation, cells were treated with Nano Glo HiBiT Lytic Detection System (Promega) to detect the HiBiT-BRD4-derived luminescent signals with EnVision Xcite (PerkinElmer). The number of cells in each well was estimated based on DNA content measured using CellTox Green Reagent (Promega) with EnVision Xcite and the luminescent intensity of HiBiT-BRD4 was normalized with the fluorescent signals of CellTox Green Reagent. The BRD4 expression levels in BET protein degrader-treated cells were calculated as the percentage of those in DMSO-treated cells.

### Statistical analysis

Statistical analyses using unpaired two-tailed Student’s t-test were performed with KaleidaGraph software (Synergy Software).

## Supplementary information

Supplementary information.

Supplementary Movie S1.

Supplementary Movie S2.

Supplementary Movie S3.
