# Supplementary material for: Characterization of cereblon-dependent targeted protein degrader by visualizing the spatiotemporal ternary complex formation in cells
Source: Sci Rep. 2020 Feb 20;10:3088. doi: 10.1038/s41598-020-59966-5 (PMC7033280; doi:10.1038/s41598-020-59966-5)
Supplement: Supplementary file 1 — Supplementary information. [file 41598_2020_59966_MOESM1_ESM.pdf]

**Supplementary information**

# Characterization of cereblon-dependent targeted protein degrader by visualizing the spatiotemporal ternary complex formation in cells

Tomohiro Kaji*, Hiroshi Koga, Mutsumi Kuroha, Toshihiko Akimoto and Kenji Hayata

Biochemistry Research Group, Biological Research Department, Daiichi Sankyo RD Novare Co., Ltd., 1-16-13 Kitakasai, Edogawa-ku, Tokyo 134-8630, Japan

*Corresponding author: Tomohiro Kaji, Ph.D.

Biochemistry Research Group, Biological Research Department, Daiichi Sankyo RD Novare Co., Ltd., 1-16-13 Kitakasai, Edogawa-ku, Tokyo 134-8630, Japan

Tel.: +81-3-5696-8301

e-mail: kaji.tomohiro.xc@rdn.daiichisankyo.co.jp

**Supplementary Figure S1**

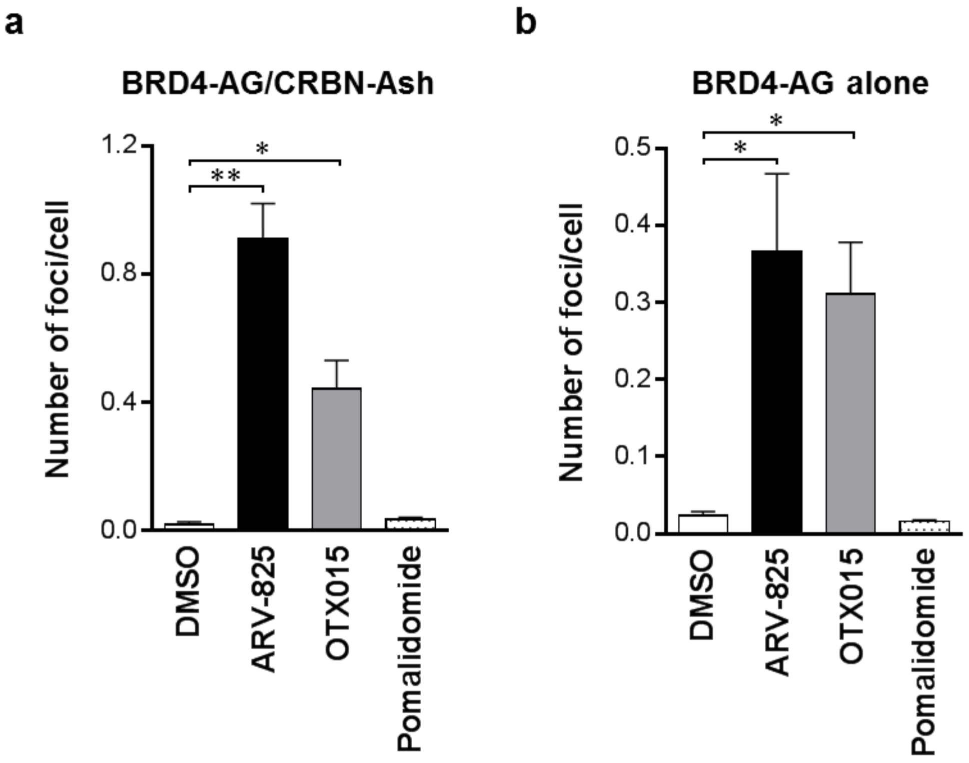


**Supplementary Figure S1 Azami-Green-fused BRD4 formed foci with its binders in the absence of Ash-tagged CRBN.**

293A cells were coexpressed with BRD4-AG and CRBN-Ash (a) or expressed with BRD4-AG alone (b). Cells were treated with 1 μM ARV-825, OTX015 and pomalidomide in 0.5% DMSO for 30 min. The number of foci per cell was quantified as in Fig. 1b. Data shown are the mean (n = 4) + S.D. and are representative of two independent experiments. *$p < 0.01$, **$p < 0.001$.

**Supplementary Table S1. Schematic representation of a workflow for evaluating CRBN-dependent TPD molecules with the Fluoppi system.**

| Step | Workflow | Details |
|---|---|---|
| 1 | Vector construction | ・ To generate a total of 8 kinds of plasmid vector<br>✓ Target gene plasmid vector: AG- or Ash-tagged at N- or C-terminus (4 types)<br>✓ CRBN plasmid vector: AG- or Ash-tagged at N- or C-terminus (4 types) |
| 2 | Determination of vector combination | ・ To transfect adherent cells with the same amount of AG and Ash vectors<br>✓ All combinations of vectors: 8 ways<br>・ To determine the best combination of vectors in terms of:<br>✓ Clear foci formation induced by TPD molecules<br>✓ Simple dose-dependence studies of TPD molecules<br>✓ No formation of foci in a steady state |
| 3 | Determination of vector ratio | ・ To determine the appropriate ratio between AG and Ash vectors<br>✓ Clear foci formation induced by TPD molecules<br>✓ Appropriate fluorescence expression<br>✓ No formation of foci in the treatment with a moiety of TPD molecules |
| 4 | Evaluation of TPD molecules | ・ To evaluate the TPD molecules' capacities of foci formation with fixed cells in terms of:<br>✓ Dose-dependence studies of foci formation<br>✓ Kinetic studies of foci formation<br>✓ Determination of foci localization<br>・ To evaluate the early ternary complex formation with live-cell imaging |
| Remarkable features | | ・ Completely resistant to conventional fixation procedure<br>・ Large dynamic assay range<br>・ Simple and easy procedure<br>✓ Quantifiable in fixed cell samples<br>✓ Large-scale image analysis<br>✓ Robust technique suitable for drug screening assays |

**Supplementary Movies S1–S3 Live-cell imaging of ARV-825-induced foci formation.** Live-cell imaging was performed as in Fig. 4. Time-lapse movies of AG (green) and Hoechst 33342 (blue) were recorded for 1 h with 45-s intervals after the addition of 0.05% DMSO (movie S1) and 0.1 (movie S2) or 0.01 µM ARV-825 (movie S3). The delay between the injection of DMSO or ARV-825 and the first image acquisition (0 min) was approximately 80 s. Data shown are representative of three independent experiments. Scale bar in each movie indicates 20 µm.
